# Human Depotentiation following Induction of Spike Timing Dependent Plasticity

**DOI:** 10.3390/biomedicines6020071

**Published:** 2018-06-18

**Authors:** Nicole Pedroarena-Leal, Larissa Heidemeyer, Carlos Trenado, Diane Ruge

**Affiliations:** 1 Department of Psychology and Neurosciences, Translational Neuromodulation Unit, Leibniz Research Centre for Working Environment and Human Factors, Technical University Dortmund, 44139 Dortmund, Germany; nicole.pedroarena.13@ucl.ac.uk (N.P.-L.); trenado@ifado.de (C.T.); 2 UCL-Institute of Neurology, University College London (UCL), Queen Square, London WC1N 3BG, UK; larissa.heidemeyer.13@ucl.ac.uk; 3 Institute of Clinical Neuroscience and Medical Psychology, Medical Faculty, Heinrich Heine University, 40225 Düsseldorf, Germany

**Keywords:** learning, neuroplasticity, cortical plasticity, memory formation, depotentiation, disease, translational

## Abstract

Depotentiation (DP) is a crucial mechanism for the tuning of memory traces once LTP (Long Term Potentiation) has been induced via learning, artificial procedures, or other activities. Putative unuseful LTP might be abolished via this process. Its deficiency is thought to play a role in pathologies, such as drug induced dyskinesia. However, since it is thought that it represents a mechanism that is linked to the susceptibility to interference during consolidation of a memory trace, it is an important process to consider when therapeutic interventions, such as psychotherapy, are administered. Perhaps a person with an abnormal depotentiation is prone to lose learned effects very easily or on the other end of the spectrum is prone to overload with previously generated unuseful LTP. Perhaps this process partly explains why some disorders and patients are extremely resistant to therapy. The present study seeks to quantify the relationship between LTP and depotentiation in the human brain by using transcranial magnetic stimulation (TMS) over the cortex of healthy participants. The results provide further evidence that depotentiation can be quantified in humans by use of noninvasive brain stimulation techniques. They provide evidence that a nonfocal rhythmic on its own inefficient stimulation, such as a modified thetaburst stimulation, can depotentiate an associative, focal spike timing-dependent PAS (paired associative stimulation)-induced LTP. Therefore, the depotentiation-like process does not seem to be restricted to specific subgroups of synapses that have undergone LTP before. Most importantly, the induced LTP seems highly correlated with the amount of generated depotentiation in healthy individuals. This might be a phenomenon typical of health and might be distorted in brain pathologies, such as dystonia, or dyskinesias. The ratio of LTP/DP might be a valuable marker for potential distortions of persistence versus deletion of memory traces represented by LTP-like plasticity.

## 1. Introduction

Different methods of delivering magnetic stimulation can lead to shorter or more prolonged changes in synaptic excitability (neuroplasticity induction). One such method is transcranial magnetic stimulation (TMS). The availability of TMS to study mechanisms of brain reorganization is useful due to the technique’s ability to focally stimulate cortical regions such as the motor cortex and quantify the response from contralateral hand muscles. This allows for the noninvasive evaluation of changes in cortex excitability at the system level in humans [1].

### 1.1. Studying Physiological Mechanisms of Plasticity in Animal Models

The use of TMS and accompanying protocols to study plasticity mechanisms in humans is based largely on animal work. Initially, animal experiments delivered high-frequency trains of electrical stimuli to the Schaffer collateral/commissural fibers projecting from CA3 to CA1 pyramidal neurons, ensuring sufficient synaptic input would occur in order to produce post-synaptic action potentials (APs). Later studies also use intracellular recordings to pair depolarization of postsynaptic cells with afferent stimulation. The use of such simultaneous stimulation suggests that correspondence between pre and postsynaptic activity of a cell is crucial for induction of long term potentiation (LTP) [2,3], one chemical synaptic transmission process that is thought to underlie learning and memory functions [4].

It has been reported that LTP lasting for several weeks [5] is induced via the use of short bursts of 100 Hertz (Hz) stimulation at intervals of 170 [6] and 200 ms [5,7]. LTP has also been evoked by burst stimulation on the positive phase of oscillatory theta rhythm in rats [8], with comparable findings reported for CA1 hippocampal slices bathed in carbachol (cholinergic agonist) to stimulate an oscillatory theta rhythm. This preparation, in conjunction with delivery of single pulses timed to a positive theta phase, induces LTP [9].

Animal work has allowed for hypotheses postulating molecular mechanisms of synaptic plasticity. For different fibers in various areas of the brain, molecular mechanisms of synaptic plasticity underlying for example LTP and depotentiation (DP) may vary from synapse to synapse. For example, the mossy fibre-CA3 pyramidal cell synapse of the hippocampus does not require *N*-methyl-d-aspartate (NMDA) receptor activation for induction of LTP [10,11].

DP is the salvaging of synapses that have undergone LTP [12]. Physiologically, it can be considered the reversal of irrelevant/excessive potentiation. DP is considered the reverse of LTP (return to baseline) following potentiation [4,13]. Methodologically, DP is a mechanism by which changes in LTP-plasticity induced by previous protocols are successfully abolished by a second protocol, which on its own is ineffective [12]. Consequently, for instance, a high frequency conditioning protocol inducing LTP would then be erased by the application of a second protocol consisting of a short period of low frequency stimulation [13,14,15].

Reversal of LTP can be achieved via the use of trains of low frequency stimulation when administered shortly after LTP induction. Also, continuous trains of single pulses between 1–5 Hz can induce DP in animals [16,17,18]. Furthermore, 5-Hz stimulation can depotentiate LTP induced in the CA1 region of the hippocampus in vitro [19]. The efficacy of DP decreases as the time interval between LTP induction and 5 Hz stimulation is increased [19]. This suggests that there is a short time interval after LTP induction that allows depotentiation.

Although the molecular mechanisms subserving DP are not yet well defined, DP may ensure that the central nervous system is not saturated by LTP or learning, by providing a mechanism through which “forgetting” can be mediated [4]. Anomalous behavior and reversibility of LTP has been described in animal models of Parkinson’s disease (PD), drug induced dyskinesia [20], and dystonia [21], as well as Huntington’s disease (HD) [22], providing examples of similarities between animal models and humans and, therefore, suggesting a translational use for such animal models.

### 1.2. Translation of Animal Model of Plasticity to Humans

In vitro studies of synaptic plasticity in humans are limited, yet experiments looking at excisions of hippocampal tissue from temporal lobe surgery in epilepsy patients are comparable to experiments conducted in animal models [23,24], with similar molecular features of LTP demonstrated in temporal cortex [24] and the perforant path–granule cell synapses of the hippocampal dentate gyrus [23]. The latter study has shown that LTP can be induced in slices when brief tetanic stimulation is applied to perforant path fibers, with potentiated responses sustained for up to 2 hours (h). A sustained potentiation has also been observed when forskolin, a cyclic adenosine monophosphate (cAMP) agonist, is perfused onto slices, indicating an involvement of cAMP dependent signaling pathways for LTP in humans [23]. Additionally, when d-(2)-2-amino-5-phosphonovaleric acid (APV), an NMDA receptor blocker, is given during tetanic stimulation, LTP induction is prevented [24]. This supports that LTP is NMDA dependent, demonstrating links between certain animal and human studies [25,26,27].

Paired associative stimulation (PAS) and Thetaburst stimulation (TBS) are TMS (transcranial magnetic stimulation) stimulation protocols designed to generate synaptic long-term potentiation and long-term depression in humans at a systems level. They differ in terms of their focality and associativity: PAS induces plasticity via phasic suprathreshold stimulation with short pulses, i.e., repetitive suprathreshold activation of neurons. TBS has less focal effects on synapses than that of PAS and does not induce associative plasticity. On the other hand, it induces cortical excitability changes via rhythmic (Theta-rhythm) suprathreshold activation of cortical neurons similarly to paired associative stimulation. PAS is focal and restricted to specific synaptic subgroups, which are targeted by both PAS components, the peripheral nerve afferents (via electrical stimulation), and the (motor) cortical TMS pulse. It thereby activates the connections between somatosensory and motor cortices via suprathreshold induction of action potentials [28]. Human TBS [29] was developed based on plasticity-inducing protocols in brain slice preparations. TBS-plasticity is also synaptically driven but is neither focal nor restricted to specific subgroups of synapses. PAS-plasticity is associative and time-dependent, whilst the high-frequency rhythmic stimulation of TBS is believed not to induce associative plasticity.

Spike timing dependent plasticity: PAS-induced plasticity is a form of associative plasticity that in humans is generated by repeatedly pairing an electrical stimulation induced peripheral input from the median nerve afferent pathway with a TMS stimulus applied over the (motor) cortex. The inter-stimulus interval (ISI) of usually 10–25 ms is crucial for the induced effects (increased or decreased excitability) of this spike timing dependent plasticity at the system level. The effect outlasts stimulation and represents a Hebbian-like LTP or LTD. It is topographically specific which is reflected by the absence of potentiated Motor Evoked Potentials (MEPs) for muscles under the control of neighboring regions of the motor cortex that are receiving TMS but not peripheral stimulation [28,30].

Depotentiation: In order to induce DP, LTP induction can be followed by a second protocol that, when applied on its own, has no effect [12]. It has been shown that the ability to reverse LTP is greatest if the DP protocol is applied immediately after the initial plasticity inducing protocol, as the effect of DP becomes gradually less with longer intervals between both protocols [15,31,32], indicating LTP consolidation over time [12]. Repetitive rTMS involves regularly repeated stimulation of the cortex by a train of magnetic pulses [33]. DP protocols in humans have previously made use of rTMS to apply variants (i.e., shorter than usual) of continuous Thetaburst-stimulation (cTBS) for reversibility of LTP inducing protocols [12].

### 1.3. Role of Human Model of Plasticity

Animal models, both in vitro [25,26,34] and in vivo [27,35], have allowed preliminary protocols of both LTP and DP to be examined and translated to humans.

Synaptic plasticity has been studied non-invasively in humans in vivo [12,28,29,30]. Bidirectional modifications of synaptic strength elicited via periods of stimulation, for both LTP and DP inducing protocols, can be achieved using TMS. Thus, the strength of connections between neurons can now be modulated to study mechanisms of activity dependent synaptic plasticity directly in humans, allowing storage.

Depotentiation is a crucial mechanism for the tuning of memory traces once LTP has been induced via learning, artificial procedures or other activities. Putative unuseful LTP is abolished via this process. Its deficiency is thought to play a role in pathologies, such as drug induced dyskinesias. However, since it is thought that it represents a mechanism that is linked to the susceptibility to interference during consolidation of a memory trace it is an important process to consider when therapeutic interventions, such as psychotherapy, are administered. Perhaps a person with an abnormal depotentiation mechanism is prone to lose learned effects very easily, or on the other end of the spectrum, is prone to overload with previously generated LTP. Perhaps this process partly explains why some disorders and/or patients are extremely resistant to therapy.

### 1.4. Objectives

The present study seeks to identify possible depotentiation in the human brain by using TMS. PAS was employed to excite the cortex and induce LTP. Abolishment of the effects of LTP was then quantified by applying a short period variant of high frequency cTBS to induce DP.

Specific aims include (1) to answer the question: Is a nonfocal stimulation protocol like Thetaburst Stimulation in humans able to induce depotentiation of spike timing dependent LTP (induced by PAS)? (2) Determining the robustness of the modified cTBS protocol for DP following spike timing dependent plasticity induction via PAS. (3) Establishing whether the effects of each protocol change over time. (4) Determining the relationship between maximum effect of LTP and maximum change induced by the DP protocol at the individual level. Implications as to the potential application of TMS protocols for determining whether individuals would be responders to specific treatment will be discussed.

## 2. Experimental Session

### 2.1. Participants

22 healthy nonmedicated (10 women, 12 men), aged 22–45 years (mean 28.04, STD = 8.2), right handed subjects gave their informed consent prior to participation. The experiments were performed with the approval of the Ethics Committee for the National Hospital of Neurology and Neurosurgery and the Institute of Neurology Joint Research Committee (code: 03/N018, 25 February 2005). All subjects were naive to the effects of TBS and unaware of the differences between PAS and cTBS.

### 2.2. Recording and Stimulation

Subjects were seated in a chair and electromyography (EMG) recordings were taken using silver/silver chloride (Ag–AgCl) disc surface cup electrodes from the right Abductor Pollicis Brevis muscle (APB) established as the target muscle. EMG activity was recorded with a gain of 1000 and filtered with a band-pass filter (3 Hz to 2 kHz) through Digitimer D360 amplifiers (Digitimer Ltd., Welwyn Garden City, UK). Signals were recorded with a sampling rate of 5 kHz, visualized during the recording via a standard PC running Signal 5.07 Software (Cambridge Electronic Design Ltd., Cambridge, UK) and stored on a personal computer for later analysis by custom made software. Trials in which the target muscle was not relaxed were rejected offline.

**Figure 1:**
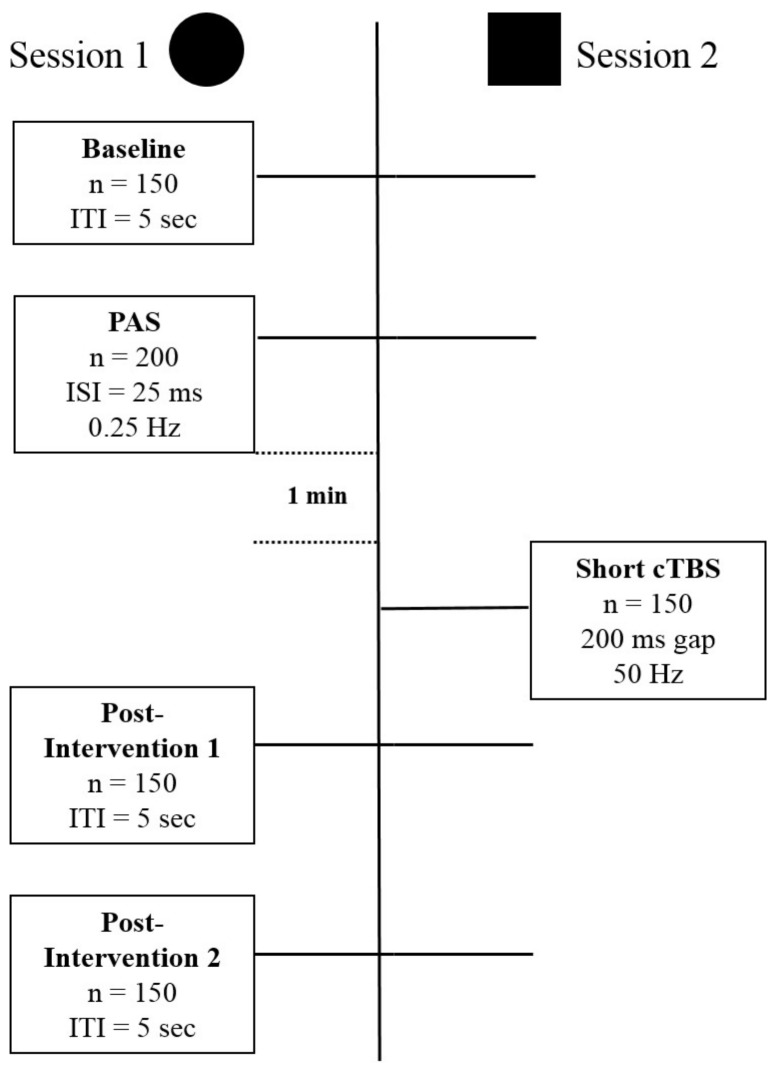
The figure schematically depicts the structure of the experiments with the different stimulation and recording components of Session 1, Long Term Potentiation (LTP)-induction, and Session 2, depotentiation-induction. Session 1 and 2 express different types of stimulation protocols, and do not reflect the order in time. Abbreviations: *n* = number, ITI = Intertrial interval, ISI = Interstimulus interval, Hz = Hertz, PAS = Paired Associative Stimulation, cTBS = continuous Thetaburst Stimulation.

Magnetic stimulation was given using a hand-held figure-of-eight coil with loop diameters of 70 mm (Magstim Co., Whitland, Dyfed, UK). Single pulse TMS was delivered by a Magstim 2000 device and TBS was delivered using a Magstim Rapid2 stimulator. Stimulation was delivered over the motor hand cortical area with the coil tangential to the scalp and the handle pointing in the posterior direction at approximately 45° angle. The motor hand area was defined as the location on the scalp where magnetic stimulation produced the largest MEP from the contralateral APB when the subject was relaxed (hot-spot). The experiment was divided into two sessions administered at least two days apart, with all participants completing both. In all sessions, the intensity of stimulation for MEP assessment was set to that required to produce a MEP of approximately 1 mV in the baseline condition. Figure 1 summarizes the design of the study. Session type 1 includes the baseline recording of 150 Motor Evoked Potentials (MEPs), followed by the Paired Associative Stimulation (PAS) procedure to induce Long Term Potentiation (LTP)-like plasticity. After a short break, this is followed by blocks of 150 MEPs to quantify the effects of the plasticity inducing intervention. Session type 2 differs from type 1 in the way that a shorter than usual modified continuous Thetaburst stimulation (cTBS), which on its own has no effect on the excitability of the stimulated area, was given after the LTP-like plasticity induction intervention. Session types 1 and 2 were given in randomized counterbalanced order. At least 48 h have been kept between sessions.

#### 2.2.1. TMS

Posterior–anterior (PA) directed currents were produced by the figure-of-eight coil held posterolaterally at an angle of about 45° to the midline. The coil was systematically moved with currents at 0.5 cm intervals in the anterior–posterior and mediolateral direction in order to identify the hotspot, defined as the position where the maximum and most stable MEP response was achieved. This position was marked for repositioning the coil.

#### 2.2.2. Thresholds

Baseline measurements were then taken and included: resting motor threshold (RMT), 1 mV level, and active motor threshold (AMT). RMT was defined as the minimum intensity needed to produce a MEP in 5 out of 10 consecutive trials (50%) of at least 50 microvolts (μV) [36]. The 1 mV level was defined as the TMS-intensity needed to produce an on average MEP size of 1 mV amplitude. Finally, the AMT was calculated as the minimum intensity of single pulse stimulation required to produce a MEP equivalent to 200 μV. Participants’ maximum activation was determined by having each participant maximally activate their APB on more than 5 out of 10 trials from the contralateral APB while maintaining a voluntary contraction of approximately 10 and 20% maximum AMTs [36].

### 2.3. Session Type I: Ltp Induction

#### 2.3.1. Baseline Recording (Pre-Recording)

In each participant at baseline, 150 MEPs were recorded at rest and the stimulus intensity set to evoke a stable MEP of approximately 1 mV at an intertrial-interval (ITI) of approximately 5.0 s.

#### 2.3.2. PAS

A paired associative stimulation (PAS) protocol was used in this experiment. Electrical peripheral nerve stimulation was delivered via a peripheral stimulator positioned to the right median nerve with an intensity three times above perceptual sensory threshold with a Digitimer DS7A stimulator. The perceptual sensory threshold was defined as the minimum stimulus intensity producing an individual, subjective report of sensation. Electrical stimulation was delivered 25 ms before a single TMS pulse over the contralateral (left) motor cortical representation of the right APB. TMS intensity was set at the 1 mV intensity and a rate of 0.25 Hz. PAS was performed for 200 trails in all subjects [28].

#### 2.3.3. P1 and P2 (Post-Recording)

Following PAS, two sets of 150 trial recordings of TMS pulses were conducted in order to observe the PAS effect.

#### 2.3.4. Session Type 2: DP

The procedure was identical to that of Session 1, with the exception that a variation of TBS (shortened continuous TBS), which is known to be inefficient on its own, was administered one minute following the PAS protocol. A modified (i.e., shortened) continuous TBS (cTBS) protocol, which if delivered on its own is known to be ineffective, was used to induce DP. Following the DP-inducing intervention, i.e., the variant cTBS, P1, and P2 (Post-Recordings), were conducted as in Session type 1. As in Huang et al., 2010, AMT was defined while the subject activated the target muscle with 20% maximum power. The stimulation intensity of TBS was defined in relation to the AMT of the subject at an intensity of 80% AMT and administered over the motor hot-spot.

The paradigm consists of a theta burst stimulation pattern in which 3 pulses are given at 50 Hz repeated every 200 ms. The pattern of the cTBS, as described by Huang et al., 2005, was modified as per Huang et al., 2010 specifications, which include the use of 150 pulses for 10 s rather than 600 pulses for 40 s. This variation has no quantifiable effects when given on its own [12].

Importantly, as aforementioned, the order of Session Type 1 and 2 was randomized and counterbalanced.

#### 2.3.5. Coil Orientations

Following the baseline recording, TMS with different current flows (coil orientations) was performed. Three different coil directions were used in the present study. The AMT was defined as the lowest intensity to evoke MEPs of 1 mV in more than 5 of 10 consecutive trials while subjects maintained approximately 10% contraction of the target muscle.

During Session 2, AMT was measured with AP and LM currents during mild contraction of the APB (~10% of the maximum voluntary contraction). Stimulus intensity was then set at 110% AP and 150% LM. Twenty-five pulses for AP and LM currents were applied over the hotspot to measure onset latency of MEPs. These measurements were taken over approximately 10 min. All trials were recorded.

#### 2.3.6. Statistics

Data was analyzed using SPSS version 21.0 (SPSS Inc., Chicago, IL, USA) and Statistica Enterprise for PC version 5.0 (StatSoft, Tulsa, OK, USA). A paired-samples Student *t*-test was conducted to compare the Baseline MEP amplitudes from Session 1 and Session 2. The mean latencies for the AP coil orientation and LM coil orientation and difference (AP-LM) between both were also calculated.

Two-way repeated measures ANOVA was used to examine whether both sessions (LTP and DP protocols) differed from each other, as well as the robustness of effect of both plasticity protocols, and whether the effects of each protocol changed over time as determined based on the change in MEP amplitude for both Sessions for MEPs normalized to baseline. Where appropriate, results were corrected for multiple comparisons using a post-hoc test and utilized to determine the time course over which DP developed to a significant level.

Additionally, regression analyses were completed. AP < 0.05 was considered statistically significant for all analyses. The Shapiro–Wilk test was implemented to assess normality. The Greenhouse–Geisser correction was used if necessary to correct for nonsphericity.

## 3. Results

Tests for normality resulted in normally distributed data for all data as tested for P1 (S.W. = 0.92, *p* = 0.20) and P2 (S.W. = 0.92, *p* = 0.09) of Session 1 and P1 (S.W. = 0.96, *p* = 0.20) and P2 (S.W. = 0.95, *p* = 0.41) of Session 2.

The RMT for the 22 participants (M = 54.64% maximum stimulator output, SD = 8.16, SE = 1.74) (Table 1) did not change before and after the plasticity inducing protocol for both sessions (LTP and DP), indicating that change in membrane excitability does not play a role for the observed effects.

A paired-samples Student *t*-test was conducted to compare the Baseline MEP amplitudes from Session 1 and 2. There was no significant difference in MEP amplitudes for Session 1 (M = 1.15 mV, SD = 0.33) and Session 2 (M = 1.36 mV, SD = 0.46); t(21) = −1.82, *p* = 0.08. This result indicates that the recording technique was reproducible and comparable.

The mean latencies for the AP coil orientation (M = 23.23 ms), LM coil orientation (M = 20.71 ms), and difference (AP-LM) between both (M = 2.52) were calculated (Table 2). On average, 22.65 ms for the AP coil orientation and 19.25 ms for the LM orientation are generally the values produced for these measurements [37]. Thus, the results show that the mean latency values for each orientation are comparable to established averages.

### 3.1. Effect of Session (Type of Intervention, LTP vs. LTP + DP) and Time

**Figure 2:**
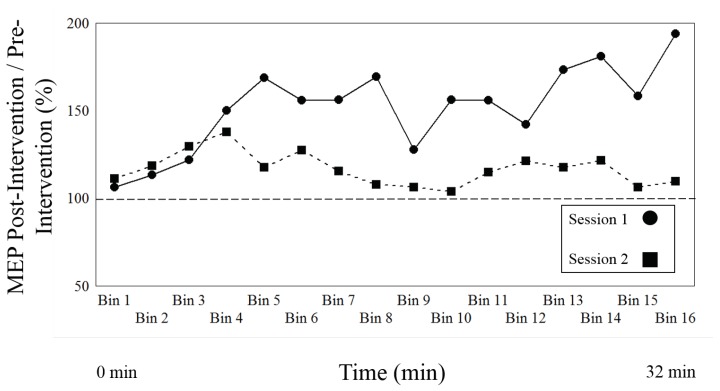
The figure shows the normalized to baseline measures (ratio pre-intervention/post-intervention) for Session type 1, LTP-induction, versus Session type 2, depotentiation induction, over time. The circles represent Session type 1, the squares Session type 2. The two lines represent the mean of all participants for each Session type.

**Figure 3:**
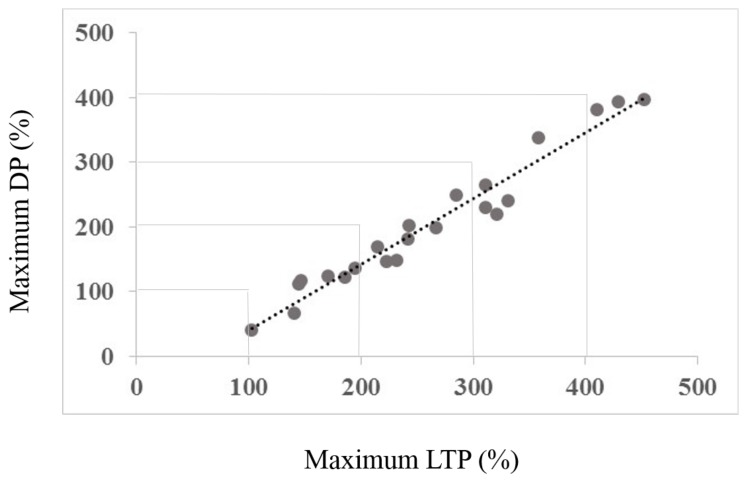
The figure shows the correlation between the maximum LTP amount and maximum change (depotentiation) amount. Note that none of the subjects shows a change towards increased LTP, all show a reduction towards baseline. Figure 4 depicts the rationale for the depotentiation quantification. The *x*-axis data derive from Session type 1, the *y*-axis data from both Session types as depicted in Figure 4.

As a primary result, Table 3 compares the change of effect between the PAS LTP inducing protocol of Session type 1 and the variant cTBS, DP eliciting, protocol of Session type 2 over the entire time course of both post induction blocks, with Figure 2 showing the mean values for all time bins. There is a main effect of session (or intervention) (F1, 21 = 7.812, *p* = 0.011), indicating that both protocols (session type 1 and 2) differ, as there is a significant change of effect between both sessions. Figure 3 shows a robust effect for both session types, as Session type 1 increases (direction of LTP) and Session type 2 demonstrates a significant decrease (direction of DP). The results also demonstrate a main effect of time (F15, 315 = 2.323, *p* = 0.004), indicating that the effects of both interventions change over time.

Finally, there was an interaction between Session type and time (F15, 315 = 2.87, *p* = 0.0003), suggesting that there are different time effects according to each intervention. Figure 2 illustrates that the LTP protocol of Session type 1 begins to increase at Level (time bin) 4 (approximately 8 min) and the DP intervention of Session 2 begins to decrease at Level (time bin) 5 (approximately 10 min).

### 3.2. Interaction between Maximum LTP and Mamixum Change

**Figure 4:**
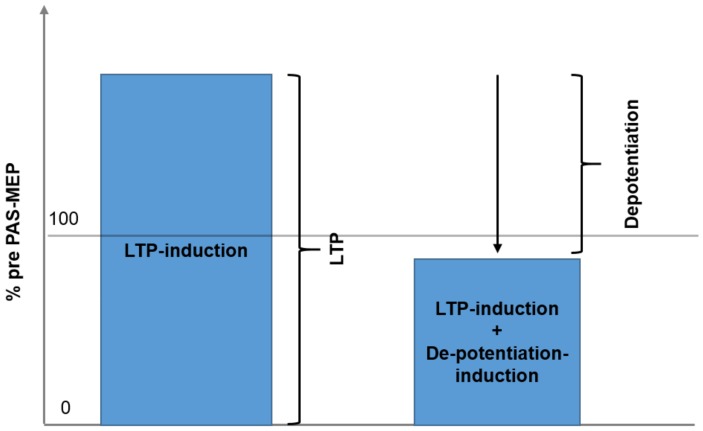
The figure demonstrates the rationale for the quantification of depotentiation. **Left** panel corresponds to session type 1: LTP is induced by PAS and quantified. **Right** panel: In session type 2 LTP is induced by paired associative stimulation and shortly after depotentiated by variant continuous thetaburst stimulation. Depotentiation is quantified by calculation result of session type 1 minus result of session type 2.

A correlation was completed to examine whether the maximum effects of Session 1 LTP inducing protocol were correlated with the maximum change elicited by the DP inducing protocol of Session 2. As a primary result, Figure 3 illustrates the regression analysis, showing a clear, significant correlation between maximum LTP and maximum change induced by the DP protocol (r = 0.980, R2 = 0.952, *p* < 0.01). Figure 4 shows the core of the depotentiation quantification: Quantified LTP (Session 1) minus quantified depotentiated LTP (Session 2) provided depotentiation-values.

## 4. Discussion

The results provide further evidence that depotentiation can be measured and quantified in humans by use of noninvasive brain stimulation techniques. They show that a nonfocal rhythmic (on its own inefficient) stimulation such as a modified thetaburst stimulation can depotentiate an associative focal spike timing dependent LTP-like plasticity. Therefore, the depotentiation-like process does not seem to be restricted to specific subgroups of synapses that have undergone LTP before. Most importantly, the induced LTP seems highly correlated with the amount of generated depotentiation in healthy individuals. This might be a phenomenon typical of health and might be distorted in brain pathologies, such as dystonia, or dyskinesias. The ratio of LTP/DP might be a valuable marker for potential distortions of persistence versus deletion of memory traces represented by LTP-like plasticity.

### 4.1. Difference of Effect on Sessions

The present findings show that there is a significant difference of effect between session type 1 and session type 2. This demonstrates in the same population of humans participating in both session types, that a spike timing-dependent type of generated LTP can be depotentiated by a thetaburst driven depotentiation protocol. As said, PAS-plasticity induction is restricted to specific subgroups of synapses, whilst modified thetaburst is nonfocal, and yet a depotentiation phenomenon is observable with this combination of stimulation protocols, as demonstrated here in humans. In animal studies looking at the interaction between LTP inducing thetabursts and DP promoting theta pulse stimulation (TPS), a continuous 5 Hz stimulus, have shown that the former leads to synaptic excitation, whereas the latter results in LTP reversal. It has been shown that LTP induction can occur by applying a pair of theta bursts with a 200 ms interval. In contrast, LTP reversal can be achieved by applying 1–30 min episodes of TPS at 5–10 Hz [15]. Stäubli & Scafidi, 1999, have also shown that TBS produces LTP in rats, as shown by an increase of up to 40% in excitatory postsynaptic potential (EPSP) slopes following TBS when compared to baseline. TPS of 300 pulses administered directly after LTP induction shows an immediate and complete reversal of potentiation [32].

LTP has also been induced using two 1-sec-long trains of 100 Hz to induce EPSPs, with 5 Hz stimulation producing pronounced reversal of synaptic transmission (DP) when administered following LTP induction [38]. Other studies have shown a difference of effect when using a tetanus of high frequency stimulation (HFS) between 100 [31,39] and 200 Hz [14], with DP reversing the effects of the first intervention when a low frequency stimulation (LFS) of 1 Hz and 2 Hz [39] or 5 Hz [14] is applied.

LTP can be induced by burst stimulation on the positive phase of theta rhythm in rats [8]. DP can be induced in animals using the negative phase of TBS [40,41], with similar results repeated for in vivo studies [35].

The rhythm induced by the DP paradigm consists of a theta burst stimulation pattern in which three pulses are given at 50 Hz repeated every 200 ms. The pattern of the cTBS, as described by Huang et al., 2005, was modified as per Huang et al., 2010 specifications, which include the use of 150 pulses for 10 s rather than 600 pulses for 40 s. This variant makes the stimulation when given alone ineffective, representing a compromise between using too short a protocol that would induce facilitation and too long a stimulation period that causes excessive inhibition [12].

Huang et al., 2010, have used shorter forms of cTBS than conventionally employed to induce DP. Since lower intensity stimulation might not activate the same circuits as those involved in LTP, shorter protocols ensure that a higher intensity can be used safely and effectively to trigger circuits that are also involved in excitatory forms of synaptic plasticity (LTP). Specifically, DP could induce erasure of LTP via NMDA receptor activation of PP1 [13]. For this reason, 150 pulses have been implemented in this project and previous studies, as trains of TBS shorter than 75 tend to be excitatory [29].

The clear, robust difference of effect between both sessions in this particular project indicates that there are indeed two different effects induced by both interventions, with variant cTBS producing a constant approximation to baseline for all participants (DP). Additionally, these protocols show that LTP and DP differ with respect to their persistence over time, potentially indicating different underlying mechanisms [4].

The nature of the DP paradigm prompts the erasure of the STDP plasticity induced by PAS. This LTP induced by TMS in the PAS paradigm has been hypothesized to activate intracortical fibers with horizontal distribution, resulting in activation of postsynaptic pyramidal output cells. Afferent fibers also activate these cells with origins in both subcortical and cortical areas. Median nerve stimulation (somatosensory information) is thought to reach the motor cortex via corticocortical fibers from the somatosensory cortex following transmission via thalamocortical fibers from the thalamus [28].

The results also show an effect for time, with the direction of effect changing over the course of each session and building in each intervention’s indicated direction. LTP inducing session type 1 shows a continuous increase, and DP eliciting session type 2 displays a steady decrease over time following an initial LTP-like effect. Stäubli and Scafidi, 1999, reported that TPS of 300 pulses administered 30 s after LTP induction results in a persistent reversal of LTP lasting the duration of the test session. The study shows that there is an increase and progressive maintenance of DP that approximates baseline over time when using a different intervention. These results mirror the effects observed for this project, as session type 1 intervention shows maintenance over time of potentiation, following the administration of one particular intervention (LTP inducing PAS), and a progressive return to baseline over time following a DP inducing variant cTBS protocol.

### 4.2. Interaction between Maximum LTP (Induced by Session Type 1) and Maximum Change (Induced by Session Type 2)

The results indicate a significant correlation between the maximum effect induced by intervention of session type 1 (LTP induction) and the maximum change induced by intervention of session type 2 (depotentiation induction). It suggests that the amount of change prompted by the second intervention is associated with the amount of LTP that each individual shows during session type 1. The result points to the presence of interindividual variation in amount (and time course) for DP.

Since an unlimited amount of potentiation could destabilize neuronal networks [42,43,44], mechanisms such as DP might serve to control for refined network modifications. The results of this project report a significant interaction between session and time. Consequently, an examination as to the reasons underlying the dependence of maximal change on maximum LTP is facilitated by a discussion as to the potential reasons motivating the variability in persistence of LTP during session type 2.

The temporal persistence of LTP has been proposed as having underlying links to the intensity of NMDA receptor activation [45], as well as to the accompanying processes of protein synthesis [46,47]. The time frame after LTP induction has been hypothesized to represent a period where protein synthesis-independent LTP is consolidated by plasticity associated proteins [48].

This selective targeting of proteins to synapses activated during tetanic stimulation has been suggested to involve synaptic tagging [49]. Synaptic tagging indicates that plasticity proteins synthesized in the cell body as a result of dendritic activations for various different inputs (among them, glutamatergic input from tetanic stimulation as proposed in animal studies) would distribute in a somewhat nontargeted manner. At the point of LTP induction, synapses potentially flag certain proteins for sequestering. The proteins then contribute to the consolidation of LTP in an input specific manner [49].

The variance in amount of maximum change (evoked by DP inducing protocol) could also be related to these mechanistically tied arguments, as the amount of protein synthesis and quantity of NMDA receptor activation can vary between individuals and possibly contribute to the amount of change induced [50,51,52]. The importance of such variance is evident when considering that changes in the strength of synaptic connection have been proposed to have a central role in learning and memory.

Motor learning induces an increase in motor cortical excitability for animals [43,53,54] that is blocked by NMDA receptor antagonists [4]. Additionally, animal models have shown that learning engages LTP in M1 and that these mechanisms, as well as subsequent engagement of memory, are associated with a reduction, or occlusion, of LTP [42,43,44].

Cantarero et al., 2013 have assessed whether application of a DP intervention over primary motor cortex (M1) after motor learning affects LTP and retention of motor skill learning [55]. It was found that the magnitude of motor memory retention is proportional to the magnitude of LTP consolidation. Induction of DP in the M1 results in reduced learning dependent LTP and disrupts the consolidation of LTP after learning.

This result suggests that DP is involved in halting the process of learning by acting as a marker for the end of the consolidation period, permitting neuronal networks to reset and prepare for future learning dependent LTP [55]. When such a mechanism is deficient, there can be consequences for learned information. The failure to reverse LTP prevents adequate consolidation of memory, whereas deficient DP could be implicated in retention of irrelevant information and excessive DP could, on the other hand, be associated with an inability to adequately reinforce or retain memories.

Parallels can be extended to the results of this study, as the amount of maximal LTP correlates to the amount of maximal change in Session type 2 following the DP inducing intervention. Cantarero et al., 2013 supports the proposal that the amount of maximal change potentially occurs as a reset response to induced LTP. In other words, more LTP predisposes an individual to show a greater effect to the DP inducing intervention as a reflection of the amount of change that needs to occur to reset the neuronal network for future learning dependent LTP [55].

In this study, an approximation to baseline was observed in all subjects following the DP inducing intervention. Maximum change in this project corresponds to a return towards baseline, and in no case an increase in the direction of more LTP. Consequently, the definition of DP in this project, as observed in the results, can potentially be linked to the proposal of a mechanism for resetting neural network for future potentiation. Interindividual variation in DP amount could then be suggested to be associated with the amount of change needed to reset the system for future LTP dependent learning. Such a distributed variety of synaptic learning rules presented in the research and evidence discussed thus far, however, indicate that there is unlikely to be one sole explanation for interindividual variations in amount of DP.

DP induces a modification of plasticity, a change dependent on specific synaptic inputs, which can be recruited separately by the change of orientation of the TMS-coil [56]. Studies have shown that different descending volleys are elicited by single pulse TMS depending on the current flow. For example, the PA current normally elicits early indirect waves (I-waves), whereas the anterior posterior (AP) current recruits late I-waves and the lateromedial (LM) current at high stimulus intensity tends to evoke direct-waves (D-wave) [37,57].

In this study, PA-directed currents were produced by the figure-of-eight coil held posterolaterally at an angle of about 45° to the midline. This orientation was used throughout the various different measurements described thus far. AP-directed currents were elicited by placing the coil 180° to the PA currents. Finally, the coil was placed with the handle pointing leftwards for LM-directed currents (90° from midsagittal line). Concerning the relevance for the DP paradigm, the question is whether the resulting differences in correlation strength between LTP and DP-effect are induced by individual differences in mechanisms of synaptic plasticity or, rather, by populations of neurons being stimulated. The present study used two measures of latency difference: AP-LM and PA-LM. Hamada et al. established the former as a measure of efficiency of late I-wave recruitment (longer latency differences indicate higher efficiency of I wave requirement) and the latter as a measurement of more standard coil orientation stimulus (since PA is the lowest threshold activation at hand area and tends to recruit I1-waves). Correlations have been established between AP-LM latencies and differences for cTBS response, with responders showing a relationship between expected inhibition after cTBS and greater AP-LM latencies and the opposite effect for nonresponders, suggesting that interindividual variation in response to TBS plasticity protocols is due to differences in population of neurons activated by each TMS pulse rather than the paradigm itself [56].

However, the current study produces no correlation between maximum change (DP) and latency. This indicates, perhaps, that interindividual differences resulting from the DP paradigm are functions of intrinsic changes that occur as a result of neuronal unmasking. The amount of response (maximum change) may then be dependent on differences in the susceptibility of intrinsic neurons.

The fact that LTP amount and maximum change via DP-induction correlate might be a typical feature of health and future studies in patient populations might reveal distortions of these capacities.

### 4.3. Implication

Interindividual variability in both intensity and duration of LTP and DP raises questions as to the role of these mechanisms in functions such as learning. Induction methods of LTP have often proved to be long lasting but not necessarily permanent [58,59,60,61]. The impermanence of LTP likely serves a role of LTP in memory updating, flexibility, and adjustment to the current state of the individual [4].

A transient form of LTP could serve to trigger lasting modifications that themselves serve as more permanent substrates for memory. The proposal that LTP is periodically refreshed and that it serves as a transient triggering mechanism for other changes necessitates an examination as to the role of other forms of plasticity in learning and “forgetting” [62].

DP represents one such mechanism that is compatible with this view of LTP as an initial process that later gives way to other mechanisms. LTP reversal by theta rhythms suggests that if LTP serves as a memory mechanism, it must be preserved or protected from reversal until a certain point. This marker in time suggests that LTP is meant to persist only until certain neural activity is accomplished. DP then acts as a regulatory process that returns the subset of potentiated synapses to baseline activity levels, allowing other functions to either augment the detail of newly encoded information [15] or prevent a saturation of synaptic plasticity [60]. Interestingly, DP varies quite extensively among individuals in this project as well as in the mentioned studies.

Interindividual differences in DP amount could potentially have clinical implications for identification of patient response to therapy. Long-term treatment with the dopamine precursor levodopa (L-DOPA) is known to induce dyskinesia in PD patients. It has been shown that rats treated with L-DOPA showing motor improvement without dyskinesia elicit DP in response to LFS. In contrast, rats showing dyskinesia in response to L-DOPA treatment do not elicit DP [20].

The striata of dyskinetic rats was subsequently shown to contain abnormally high levels of an inhibitor of PP1, one of the proposed phosphatases involved in signaling cascades for DP [20]. Since DP is suggested to occur for reset of neuronal networks needed for future learning dependent LTP [55], the results indicate that abnormal information storage is linked to the development of L-DOPA induced dyskinesia [20]. Such abnormalities could potentially be predicted by deviations in correlations between maximum LTP and DP, as healthy individuals seem to have matched responses for both mechanisms in this study.

Such evidence for the role of synaptic plasticity abnormalities in specific disorders leads to the proposal for development of clinical tests based on DP measurements. Measurements of interindividual patient differences in DP amount can potentially play a future role in predicting patient response to treatment by indicating abnormal patterns of information storage.

## 5. Conclusions

It has been demonstrated that there is a significant induction of different effects by both interventions. Additionally, a relatively constant progression towards baseline (DP) is shown for all subjects following the session type 2 protocol. The results, however, also show variability in the amount and time course for DP, as indicated by the correlation between maximum LTP and change. This result suggests that the amount of maximum LTP is one potential factor that influences interindividual differences in amount of DP.

The study demonstrates the effectiveness of the session type 2 paradigm for DP induction as well as the variability among individuals. Such interindividual variance suggests that measures of the robustness of effect of DP, and possibly time course, can serve as potential biomarkers to predict retention and resistance to therapy, as DP is suggested to play a role in regulatory processes of information encoding and erasure. These clinical implications suggest that further investigation of DP would potentially result in markers serving as assessment for evaluation of personalized patient therapy. Such tools would perhaps not only provide a more individualized form of medical attention but, additionally, increase the efficacy and safety of treatment. Note that at the system level LTP is precisely termed “LTP-like plasticity”.

## Figures and Tables

**Table 1:** Mean resting motor threshold (RMT) values. The table summarizes the descriptive statistics for the RMT values for Session 1 for all 22 subjects.

Subject	RMT (% mso)
1	63
2	73
3	50
4	47
5	54
6	60
7	60
8	54
9	58
10	58
11	55
12	43
13	44
14	55
15	44
16	58
17	70
18	47
19	41
20	54
21	53
22	61
Mean	54.64
Standard Deviation	8.16
Standard Error	1.74

**Table 2:** ∆AP-LM latency values for correlations. The table summarizes the values used for the regression analysis for the maximum LTP effect produced by the Session 1 intervention and latency as well as the values for the correlation for maximum change and latency for all 22 subjects. Latency was calculated as the difference between the AP and LM coil direction.

Subject	AP Latency (ms)	LM Latency (ms)	Latency (ms) (Difference AP-LM)
1	21.98	17.68	4.30
2	24.25	19.85	4.40
3	24.13	21.44	2.69
4	21.28	20.02	1.79
5	22.69	19.27	3.43
6	24.50	22.31	2.19
7	24.28	22.52	1.76
8	23.36	20.61	2.76
9	24.02	20.16	3.86
10	24.39	20.57	3.82
11	24.18	22.75	1.43
12	22.71	20.67	2.05
13	22.74	20.26	2.48
14	22.03	20.35	1.68
15	21.75	20.71	1.03
16	25.08	23.53	1.56
17	22.28	19.42	2.87
18	22.52	20.20	2.32
19	22.39	21.37	1.02
20	22.83	20.18	2.65
21	24.05	22.08	1.97
22	23.17	19.70	3.47
Mean	23.23	20.71	2.52

**Table 3:** Summary of all effects: session (LTP vs. depotentiation (DP)) compared to time. The table summarizes the results for the two-way repeated measures ANOVA for Session (intervention) compared to time. The LTP inducing PAS protocol was administered during Session 1 and the DP eliciting cTBS intervention was applied during Session 2. The time course is inclusive of all Bins or levels. The table summarizes the significant main effects for session and time as well as the statistically significant result of the interaction between both variables.

Variable (1-Session, 2-Time)	df Effect	MS Effect	df	MS Error	F	*p*-Level
1	1	219684.1094	21	28121.38672	7.811994553	0.010850717
2	15	6917.164063	315	2978.043457	2.322721004	0.003729491
12	15	8113.486328	315	2825.038086	2.871991873	0.000298259
